# Safety of intravenous insulin aspart compared to regular human insulin in patients undergoing ICU monitoring post cardiac surgery: an Indian experience

**DOI:** 10.1186/s40200-015-0152-3

**Published:** 2015-04-03

**Authors:** Manoj Chawla, Harshad Malve, Harshvi Shah, Shwetal Shinde, Anil Bhoraskar

**Affiliations:** Department of Diabetology, Asian Heart Institute, G/N Block, Bandra Kurla Complex, Bandra East, Mumbai, Maharashtra 400051 India; Lead Medical for Asia Pacific region, Ferring Pharmaceuticals, 24th Floor, Sunshine Towers, Senapati Bapat Marg, Elphinstone (West), Mumbai, 400013 India

**Keywords:** CABG, Cardiac surgery, Diabetes, Hyperglycemia, Intensive care

## Abstract

**Background:**

Poor perioperative glycemic control increases risk of infection, cardiovascular accidents and mortality in patients undergoing surgery. Tight glycemic control by insulin therapy is known to yield better outcomes in such patients. Intravenous (IV) insulin therapy with or without adjunctive subcutaneous insulin therapy is the mainstay of managing hyperglycemia in perioperative period. This observational study assessed the safety of IV Insulin Aspart (IAsp) as compared to Regular Human Insulin (RHI) in patients undergone cardiac surgery at a tertiary care hospital.

**Methods:**

203 patients received IV IAsp (n = 103) and RHI (n = 100) respectively. Safety was assessed by frequency and severity of adverse events (AEs) & serious adverse events (SAEs) during hospitalization.

**Results:**

IAsp effectively controlled mean blood glucose levels to 159.87 ± 41.41 mg/dl similar to RHI (160.77 ± 44.39 mg/dl). No serious adverse event was reported. The incidence of hypoglycemia was similar in both the groups. The insulin infusion rate, time for which insulin infusion was withheld and mean blood glucose during hypoglycemia was significantly high in RHI group.

**Conclusion:**

This study has shown similar safety of IV IAsp as compared to IV RHI in the post cardiac surgery patients. However physicians preferred IAsp as it offers advantage during transition. IV IAsp offers an effective and safe option for managing hyperglycemia in patients in ICU post cardiac procedures.

## Introduction

Hyperglycemia secondary to diabetes, impaired glucose tolerance or stress-induced hyperglycemia is a common complication in hospitalized patients [1]. It is estimated that >30 to 50% hospitalized patients experience significant hyperglycemia in hospitals [2]. Uncontrolled hyperglycemia is an important predictor of adverse hospital outcomes, prolonged hospitalizations and accounts for half of all health care expenditures for hospitalized patients [3,4].

The prevalence of diabetes in India is increasing and presently it is about 63 million. It is estimated to reach around 101.2 million by the year 2030 [5]. With this increase in prevalence, India faces an epidemic of Type 2 diabetes. Thus, doctors from all fields, including Intensivists are more likely to face issues like hyperglycemia in critical care settings. Inpatient hyperglycemia is commonly associated with increased morbidity and mortality rates in hospitalized patients [6]. It is also well established that perioperative glycemic control in patients undergoing cardiac surgery reduces morbidity, infection, myocardial or vascular dysfunction and enhances long-term survival [7]. Intravenous (IV) insulin therapy with subcutaneous (SC) insulin is the mainstay of treatment in management of Hyperglycemia in hospitalized patients and has shown to improve morbidity and reduce mortality rates in critically ill patients [7,8]. Some patients can be managed safely with SC administration of insulin but for most experts IV administration is the preferred route because of the delayed onset of action and prolonged half-life of SC regular insulin [9].

Faster onset and short duration of action leading to meal time flexibility make rapid acting Insulin analogues, particularly insulin aspart, a choice for quickly achieving the target blood glucose levels [10]. Insulin aspart has demonstrated a beneficial safety/efficacy ratio in patients with Type 1 or Type 2 diabetes. When compared with short acting human insulin, it has demonstrated a better efficacy and safety profile [11]. Studies have shown that insulin aspart can be used intravenously [12].

Several clinical studies with IV infusion of short acting insulin have shown reduced morbidity and mortality in hospitalized patients with hyperglycemia [7,13-16]. Insulin aspart with a better efficacy and safety profile than short acting human insulin has been used intravenously and appears to be a good alternative to short acting human insulin [17-19]. Even though insulin aspart is approved for IV use, very limited data is available on its safety and efficacy of it in a subset of Indian patients undergoing cardiac procedures. Thus in this study we tried to provide vital information on safety and efficacy of insulin aspart as compared to regular human insulin in patients undergoing ICU monitoring post cardiac surgery. Also this study being an observational study provides the real life scenario on use in IV insulin in patients undergoing cardiac procedures.

## Methodology

Permission was taken from Institutional Ethics Committee at Asian Heart institute, Mumbai, prior to initiation of the study. In this single centre, open label, non-randomized, observational study, 203 hospitalized patients scheduled for cardiac procedure requiring IV insulin therapy, were given either regular human insulin (RHI) or Insulin Aspart (IAsp) as per the discretion of the treating physician. Physicians were free to use IV insulin infusion as per their discretion and clinical judgment. The patients were type 2 diabetes patients post-CABG (Coronary Artery Bypass Grafting) for ICU (Intensive Care Unit) monitoring. The patients who are unlikely to comply with protocol or allergic to study insulins were excluded. Contraindications for both the insulins were considered while selecting the patients. The patient was given an option to withdraw at will any time during the study duration. The stopping of intravenous insulin therapy was at the discretion of the treating physician, based upon the clinical evaluation.

Data was collected at baseline, while on IV insulin and 24 hours after cessation of IV insulin therapy. Before initiating IV insulin therapy, the physician gathered the demographic data and medical history. The blood glucose measurements (measurements spread over 24 hours), timing and dose of IV insulin, number and type of AEs & SAEs, number of minor and major hypoglycemic episodes, number of rescue dextrose infusions and time for which IV insulin was withheld due to hypoglycemia, clinical condition and laboratory parameters of patients receiving insulin 24 hours was noted. Following cessation of IV insulin blood glucose measurements (at least 10, including one at the end of 24 hours following cessation of IV insulin infusion), timing and dose of substituted subcutaneous insulin preparation, other medications administered, number of minor and major hypoglycemic episodes, number and type of AEs & SAEs were recorded. In addition treating physicians entered their comments on IV insulin therapy in a questionnaire provided to them. All adverse events reported by the patient or physician during IV insulin infusion were recorded on a separate adverse event form.

Clinically acceptable range for each subject was defined by the treating physician based on initial diagnosis and the treatment target and was broadly kept at < 140 mg/dl. Minor hypoglycemic events were defined as events with one of the following characteristics: 1) symptoms of hypoglycemia with confirmation by blood glucose measurement < 56 mg/dl (3.1 mmol/L) and which is handled by the subject himself/herself, or 2) any asymptomatic blood glucose measurement < 56 mg/dl (3.1 mmol/L). Major hypoglycemic events were defined as events with severe central nervous system symptoms consistent with hypoglycemia in which the subject is unable to treat himself/herself and has one of the following characteristics: 1) Blood glucose < 56 mg/dl (3.1 mmol/L), or 2) Reversal of symptoms after either carbohydrate intake, glucagon or intravenous glucose administration. Rescue dextrose was administered when the patient had hypoglycemic episode.

Because of the observational nature of the study mimicking “real world” scenario, any procedure ordered by the physician during this study was the one that was appropriate to the routine care delivered to the patient at the discretion of the participating physician. Patients were under direct medical care during the entire study period. Safety of IV insulin was assessed by monitoring the frequency and type of adverse events (AEs) and serious adverse events (SAEs) occurred in patients on IV insulin. Efficacy of the treatment was assessed by the time to reach and time spent within clinically acceptable range of blood glucose levels with IV insulin. Physicians were asked about the reasons for preferring the specific study insulin (IAsp or RHI) like: Quick achievement of target blood glucose levels, Very safe to use, Easy to switch from IV to SC or any other reasons.

The study was conducted in accordance with the principles of declaration of Helsinki and good clinical practice (GCP) guidelines. Before the commencement of the study, ethics committee approval and informed consent from all the participants was obtained.

### Statistical methods

Data was expressed as mean and standard deviation (SD) for continuous variables, and frequency and percentages for categorical variables. Student *t* test has been used to find the significance of study parameters. Leven’s test for homogeneity of variance has been performed to assess the homogeneity of variance. Chi-square/Fisher Exact test has been used to find the significance of study parameters on categorical scale between two or more groups.

## Results

Out of total 203 patients, 103 patients received IV IAsp and 100 patients received IV RHI. Age, height, weight, previous use of insulin therapy and diabetes duration were similar in both the groups (Tables 1 and 2). No infection or mortality was reported in the study. Physicians’ preference to use either therapy did not vary as per the baseline characteristics of the study population. 95% physicians however preferred IAsp at the end of study. Adverse events were similar in both the groups (Table 3). No serious adverse event was noted during the study. Severity of adverse events was more in RHI group as compared to IAsp group, 15 moderate level adverse events were noted in RHI group as compared to 11 in IAsp group (Table 3). The adverse events were unlikely due to the study insulin therapy.

**Table 1:** **Demographics and baseline characteristics of the overall population**

**Parameter (Unit)**	**IAsp group**	**RHI group**	**p value**
Total Patients (n)	103	100	
Male: Female	4.72: 1	5.25: 1	0.78
Mean age (Years)	61.18 ± 9.13	60.48 ± 8.52	0.57
Mean Height (cm)	165.44 ± 11.59	165.14 ± 10.91	0.85
Mean Weight (kg)	71.35 ± 13.17	71.91 ± 13.02	0.76
Insulin naïve patients (n)	80	73	0.52

Values for Age, height & weight are represented as Mean ± SD; Unpaired *t* test.

**Table 2:** **Duration of diabetes in study groups**

**Diabetes duration (years)**	**IAsp group**		**RHI group**	
	**n**	**%**	**n**	**%**
<1 years	20	19.4	29	29.0
1–5 years	29	28.2	16	16.0
5–10 years	25	24.3	19	19.0
11–20 years	20	19.4	17	17.0
>20 years	9	8.7	19	19.0
Total	103	100.0	100	100.0

Data represented as percentages. Samples are gender matched with p = 0.778.

**Table 3:** **Severity of adverse events**

**Severity**	**IAsp group**		**RHI group**	
	**n**	**%**	**n**	**%**
Mild	34	75.6	29	65.9
Moderate	11	24.4	15	34.1
Severe	0	0.0	0	0.0
Total	45	100.0	44	100.0

Data represented as percentages.

There was no significant difference in 2 groups for hypoglycemic episodes. However insulin infusion rate, the time for which insulin infusion was withheld and mean blood glucose during hypoglycemic episodes was significantly high in RHI group as compared to IAsp group (Table 4). The amount of dextrose infused as rescue medication was significantly high in IAsp group (Table 4). Mean blood glucose while the patients were scheduled for cardiac surgery was 142.42 ± 33.05 mg/dl and 141.94 ± 37.13 mg/dl in IAsp and RHI group respectively and was not significantly different. The patients were started on IV insulin therapy and at the cessation of IV insulin therapy the mean blood glucose was safely controlled by IAsp (159.87 ± 41.41 mg/dl) and RHI (160.77 ± 44.39 mg/dl) (Table 5). Time to reach and time spent within clinically acceptable range of blood glucose levels were similar in both the groups.

**Table 4:** **Comparison of hypoglycemic episode in two groups of patients**

**Hypoglycemic episode**	**IAsp group (n = 103)**	**RHI group (n = 100)**	**p value**
Minor episode	3	0	0.086
Major episode	5	3	0.497
Mean Insulin Infusion Rate (ml/hr)	0.44 ± 0.46	1.17 ± 1.15	0.0001^*^
Mean Blood Glucose (mg/dl)	55.67 ± 8.63	71.00 ± 8.18	0.0001^*^
Mean time for insulin infusion withheld (mins)	83.3 ± 36.06	140.0 0 ± 38.56	0.0001^*^
Mean Rescue dextrose infused (Grams)	9.05 ± 3.27	6.25 ± 0.00	0.0001^*^

All values represent Mean ± SD; Unpaired *t* test: ^*^p < 0.001.

**Table 5:** **Comparison of Glucose levels**

**Glucose levels**	**IAsp group (n = 103)**	**RHI group (n = 100)**	**p value**
Before Switch to IV	142.42 ± 33.05	141.94 ± 37.13	0.515
After switch to IV	159.87 ± 41.41	160.77 ± 44.39	0.709

All values represent Mean ± SD; Unpaired *t* test.

## Discussion

Current guidelines recommends target Blood Glucose (BG) level at 140–180 mg/dL in ICU [20]. In the present study, Insulin aspart effectively lowered mean blood glucose to target range and was found to be as safe and effective as regular human insulin. And as results indicated the insulin infusion rate needed was significantly less with IAsp as compared to RHI. Intravenous Insulin therapy is considered the best approach for managing hyperglycemia in critical patients but once patient starts oral feeds bolus insulin is required and therefore it is essential to switch the patients to subcutaneous insulin therapy. During transition from IV to SC insulin, basal dose needs to be reduced by 20–33% as the stress hyperglycemia is reduced and dose has to be adjusted accordingly [20]. Patients with only stress hyperglycemia are subjected to a gradual reduction in the dose of their IV insulin and finally stopped. Before discharge the patients with hyperglycemia are stabilized and usually do not require insulin therapy. Udwadia *et al.* has studied use of IV Insulin aspart and found it to be safe & effective option for management of hyperglycemia in hospitals [19]. Most physicians (98.6%) expressed a preference to use IAsp in the future owing to rapid achievement of target BG, positive safety profile and convenience of shifting from IV to SC administration [19]. The findings of the present study were consistent with the study of IV Insulin aspart by Udwadia *et al.* and has shown better physician preference for IAsp. In the present study we also found more physician preference with IAsp. Also in the present study the time for insulin infusion withheld was significantly less with IAsp indicating better recovery from hypoglycemic episodes.

The use of IAsp has no benefit when used intravenously alone however it is known to be beneficial when given with SC route. The beneficial effect of IAsp in hospitals is seen once patients are transferred to subcutaneous insulin therapy as there is no need to change insulin while shifting the patient from IV to SC insulin therapy to get the advantages with SC insulin analog therapy. And this avoids any loss or wastage of insulin which may occur while changing the insulins. Patient can continue with the same insulin throughout the treatment i.e. before, during & after the hospitalization or surgery. Rapid acting analogs with their peculiar pharmacokinetic actions of fast absorption and dispersion from the subcutaneous site of injection offer rapid lowering of BG levels. Another important benefit is the meal time flexibility [21,22]. This advantage is particularly important in hospitalized and critically ill patients who do not have a predictable meal pattern. Patients on parenteral medical nutrition therapy also benefits with use of rapid acting insulins like insulin aspart as it gives better glycemic control without causing hypoglycemia [23].

This study being an observational study, has inherent limitations and confounding factors, such as a lack of tightly controlled patient populations, no control groups and susceptibility to bias. We tried to overcome the bias for control group by adding RHI group. This study has not focused on the data related to the cost-effectiveness of the study insulins. Another limitation of this study due to its observational design is the lack of a defined IV insulin infusion protocol which mimics the real life scenario however the validated protocols with demonstrated efficacy and safety are available [24]. In this study, use of insulin was at the physician’s discretion and they were allowed to choose the IV insulin protocol, which might have affected the outcomes in some patients. Use of Insulin aspart with a standardized protocol in hospitals is studied by Bernard *et al.* and found to lower the BG safely in the Emergency Department without prolonging Length of Stay (LOS) [25]. The protocol was termed as Rush Emergency Department Hyperglycemia Intervention (REDHI) protocol [25].

Use of insulin aspart by IV route was earlier studied by Robinson *et al.* in a hyperinsulinemic hypoglycemic clamp study and found that soluble human insulin and insulin aspart had similar effects upon hypoglycemia-induced alterations in cardiac repolarization [17]. IV insulin aspart with insulin detemir was studied by Dungan *et al.* to determine whether an insulin algorithm could be used in a similar manner in the setting of diabetes and stress hyperglycemia following cessation of IV (IV) insulin after cardiac surgery and they found use of IV insulin aspart to be safe in patients after cardiac surgery [26].

It is evident from earlier studies that use of insulin aspart with standard protocol in hospitals is helpful to achieve the desired glycemic control with better patient outcomes in hospitalized patients. And use of Insulin aspart is sometimes preferred to avoid change of insulin during transition. However more data is needed to compare the benefits of IV insulin aspart over regular human insulin in such patients. Present study has shown IV Insulin Aspart to be safe and effective option for managing perioperative hyperglycemia in patients undergoing cardiac procedure however there was no significant difference in safety profiles of the study insulins.

Although it is known that there are no advantages of insulin analogs when used intravenously, it will be imperative to have a comprehensive pharmacoeconomic evaluation comparing the cost-effectiveness with use of insulin aspart as compared to regular human insulin in the hospital settings. This will enable the clinicians to take the rational decisions and will give the cost-effective treatment option to the patients.
